# 4-Methyl-1-(3-pyridyl­methyl­idene)thio­semicarbazide

**DOI:** 10.1107/S1600536810048853

**Published:** 2010-11-27

**Authors:** Rongchun Li

**Affiliations:** a Department of Chemistry, Dezhou University, Dezhou 253023, People’s Republic of China

## Abstract

All the non-H atoms of the title compound, C_8_H_10_N_4_S, lie on a crystallographic mirror plane and an intra­molecular N—H⋯N hydrogen bond helps to stabilize the mol­ecular conformation. In the crystal, mol­ecules are linked through inter­molecular N—H⋯N hydrogen bonds, forming zigzag *C*(7) chains along the *a* axis.

## Related literature

For background to Schiff bases derived from thio­semicarbazone and its derivatives, see: Casas *et al.* (2001 ▶); Beraldo *et al.* (2001 ▶); Jouad *et al.* (2002 ▶); Swearingen *et al.* (2002 ▶). For bond-length data, see: Allen *et al.* (1987 ▶). For similar structures, see: Selvanayagam *et al.* (2002 ▶); Karakurt *et al.* (2003 ▶); Bernhardt *et al.* (2003 ▶); Sampath *et al.* (2003 ▶).
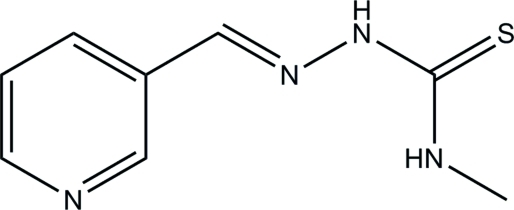


## Experimental

### 

#### Crystal data

C_8_H_10_N_4_S
*M* *_r_* = 194.26
Monoclinic, 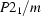

*a* = 7.276 (3) Å
*b* = 6.581 (2) Å
*c* = 10.297 (3) Å
β = 92.997 (2)°
*V* = 492.4 (3) Å^3^
*Z* = 2
Mo *K*α radiation
μ = 0.29 mm^−1^
*T* = 298 K
0.17 × 0.15 × 0.15 mm

#### Data collection

Bruker APEXII CCD diffractometer
Absorption correction: multi-scan (*SADABS*; Sheldrick, 2004 ▶) *T*_min_ = 0.953, *T*_max_ = 0.958
3208 measured reflections
1106 independent reflections
640 reflections with *I* > 2σ(*I*)
*R*_int_ = 0.041

#### Refinement

*R*[*F* ^2^ > 2σ(*F* ^2^)] = 0.043
*wR*(*F* ^2^) = 0.118
*S* = 1.02
1106 reflections
84 parameters
2 restraints
H atoms treated by a mixture of independent and constrained refinement
Δρ_max_ = 0.14 e Å^−3^
Δρ_min_ = −0.20 e Å^−3^

### 

Data collection: *APEX2* (Bruker, 2004 ▶); cell refinement: *SAINT* (Bruker, 2004 ▶); data reduction: *SAINT*; program(s) used to solve structure: *SHELXS97* (Sheldrick, 2008 ▶); program(s) used to refine structure: *SHELXL97* (Sheldrick, 2008 ▶); molecular graphics: *SHELXTL* (Sheldrick, 2008 ▶); software used to prepare material for publication: *SHELXTL*.

## Supplementary Material

Crystal structure: contains datablocks global, I. DOI: 10.1107/S1600536810048853/hb5755sup1.cif

Structure factors: contains datablocks I. DOI: 10.1107/S1600536810048853/hb5755Isup2.hkl

Additional supplementary materials:  crystallographic information; 3D view; checkCIF report

## Figures and Tables

**Table 1:** Hydrogen-bond geometry (Å, °)

*D*—H⋯*A*	*D*—H	H⋯*A*	*D*⋯*A*	*D*—H⋯*A*
N4—H4⋯N2	0.90 (2)	2.14 (3)	2.585 (4)	109 (2)
N3—H3⋯N1^i^	0.90 (1)	2.09 (1)	2.989 (3)	176 (3)

Symmetry code: (i) 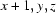.

**Fig. 1.**
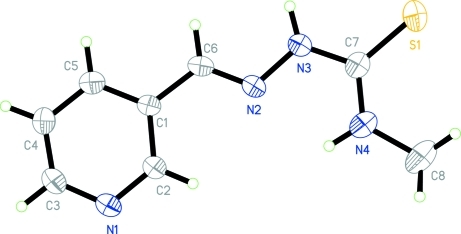
The molecular structure of the title compound, showing 30% probability displacement ellipsoids.

**Fig. 2.**
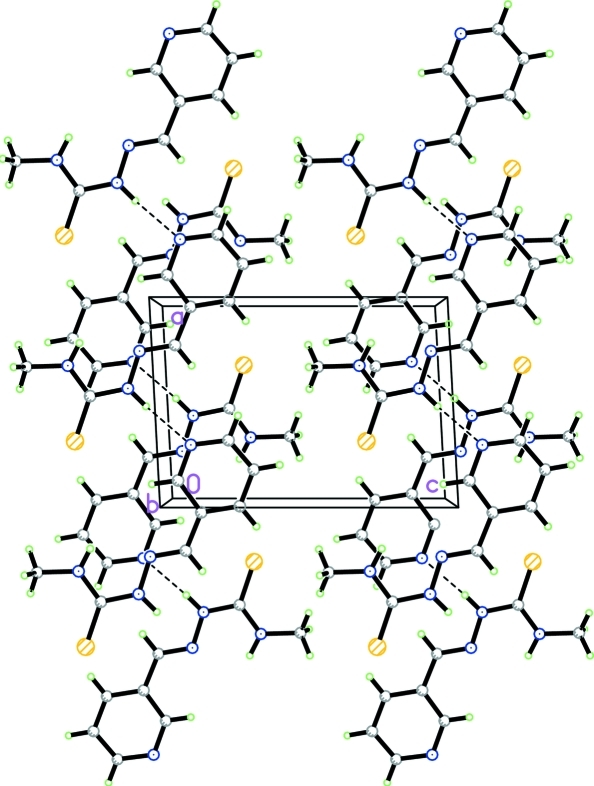
The crystal packing of the title compound, viewed along the b axis.

## supplementary crystallographic information

### Comment

Thiosemicarbazone and its derivatives are important materials for the preparation of Schiff bases (Casas *et al.*, 2001; Beraldo *et al.*, 2001; Jouad *et al.*, 2002; Swearingen *et al.*, 2002). In this paper, the title new Schiff base compound derived from the condensation of 3-formylpyridine with 4-methylthiosemicarbazone is reported.

The molecule of the title compound, Fig. 1, possess a crystallographic mirror plane symmetry. The bond lengths have normal values (Allen *et al.*, 1987), and are comparable to those observed in similar compounds (Selvanayagam *et al.*, 2002; Karakurt *et al.*, 2003; Bernhardt *et al.*, 2003; Sampath *et al.*, 2003).

In the crystal, molecules are linked through intermolecular N—H···N hydrogen bonds (Table 1), to form zigzag chains along the *a* axis (Fig. 2).

### Experimental

The title compound was prepared by the Schiff base condensation of equimolar quantities of 3-formylpyridine (0.107 g, 1 mmol) with 4-methylthiosemicarbazone (0.105 g, 1 mmol) in methanol. The excess methanol was removed by distillation. Colourless blocks were obtained by slow evaporation of an ethanol solution of the product in air.

### Refinement

The amino H atoms were located in a difference map and refined with N—H distance restrained to 0.90 (1) Å. The remaining H atoms were positioned geometrically (C—H = 0.93–0.96 Å) and refined using a riding model, with *U*_iso_(H) = 1.2*U*_eq_(C) and 1.5*U*_eq_(C8).

### Crystal data

**Table:**

C_8_H_10_N_4_S	*F*(000) = 204
*M**_r_* = 194.26	*D*_x_ = 1.310 Mg m^−^^3^
Monoclinic, *P*2_1_/*m*	Mo *K*α radiation, λ = 0.71073 Å
Hall symbol: -P 2yb	Cell parameters from 669 reflections
*a* = 7.276 (3) Å	θ = 2.7–24.5°
*b* = 6.581 (2) Å	µ = 0.29 mm^−^^1^
*c* = 10.297 (3) Å	*T* = 298 K
β = 92.997 (2)°	Block, colourless
*V* = 492.4 (3) Å^3^	0.17 × 0.15 × 0.15 mm
*Z* = 2	

### Data collection

**Table:**

Bruker APEXII CCD diffractometer	1106 independent reflections
Radiation source: fine-focus sealed tube	640 reflections with *I* > 2σ(*I*)
graphite	*R*_int_ = 0.041
ω scans	θ_max_ = 26.5°, θ_min_ = 2.8°
Absorption correction: multi-scan (*SADABS*; Sheldrick, 2004)	*h* = −9→9
*T*_min_ = 0.953, *T*_max_ = 0.958	*k* = −8→8
3208 measured reflections	*l* = −12→10

### Refinement

**Table:**

Refinement on *F*^2^	Primary atom site location: structure-invariant direct methods
Least-squares matrix: full	Secondary atom site location: difference Fourier map
*R*[*F*^2^ > 2σ(*F*^2^)] = 0.043	Hydrogen site location: inferred from neighbouring sites
*wR*(*F*^2^) = 0.118	H atoms treated by a mixture of independent and constrained refinement
*S* = 1.02	*w* = 1/[σ^2^(*F*_o_^2^) + (0.0547*P*)^2^] where *P* = (*F*_o_^2^ + 2*F*_c_^2^)/3
1106 reflections	(Δ/σ)_max_ < 0.001
84 parameters	Δρ_max_ = 0.14 e Å^−^^3^
2 restraints	Δρ_min_ = −0.19 e Å^−^^3^

### Special details

**Table:**

Geometry. All e.s.d.'s (except the e.s.d. in the dihedral angle between two l.s. planes) are estimated using the full covariance matrix. The cell e.s.d.'s are taken into account individually in the estimation of e.s.d.'s in distances, angles and torsion angles; correlations between e.s.d.'s in cell parameters are only used when they are defined by crystal symmetry. An approximate (isotropic) treatment of cell e.s.d.'s is used for estimating e.s.d.'s involving l.s. planes.
Refinement. Refinement of *F*^2^ against ALL reflections. The weighted *R*-factor *wR* and goodness of fit *S* are based on *F*^2^, conventional *R*-factors *R* are based on *F*, with *F* set to zero for negative *F*^2^. The threshold expression of *F*^2^ > σ(*F*^2^) is used only for calculating *R*-factors(gt) *etc*. and is not relevant to the choice of reflections for refinement. *R*-factors based on *F*^2^ are statistically about twice as large as those based on *F*, and *R*- factors based on ALL data will be even larger.

### Fractional atomic coordinates and isotropic or equivalent isotropic displacement parameters (Å^2^)

**Table:**

	*x*	*y*	*z*	*U*_iso_*/*U*_eq_	Occ. (<1)
S1	0.68926 (12)	0.2500	0.29184 (9)	0.0835 (4)	
N1	−0.2929 (3)	0.2500	−0.1051 (2)	0.0558 (7)	
N2	0.2508 (3)	0.2500	0.0542 (2)	0.0507 (6)	
N3	0.4324 (3)	0.2500	0.1009 (2)	0.0566 (7)	
N4	0.3217 (4)	0.2500	0.3028 (3)	0.0699 (8)	
C1	0.0350 (3)	0.2500	−0.1287 (3)	0.0496 (7)	
C2	−0.1201 (3)	0.2500	−0.0547 (3)	0.0510 (8)	
H2	−0.1020	0.2500	0.0354	0.061*	
C3	−0.3159 (4)	0.2500	−0.2351 (3)	0.0616 (9)	
H3A	−0.4353	0.2500	−0.2720	0.074*	
C4	−0.1733 (4)	0.2500	−0.3164 (3)	0.0697 (10)	
H4A	−0.1954	0.2500	−0.4061	0.084*	
C5	0.0049 (4)	0.2500	−0.2620 (3)	0.0673 (10)	
H5	0.1041	0.2500	−0.3154	0.081*	
C6	0.2213 (4)	0.2500	−0.0687 (3)	0.0581 (8)	
H6	0.3209	0.2500	−0.1217	0.070*	
C7	0.4698 (4)	0.2500	0.2318 (3)	0.0554 (8)	
C8	0.3272 (5)	0.2500	0.4451 (3)	0.0994 (13)	
H8A	0.3310	0.1125	0.4762	0.149*	0.50
H8B	0.2193	0.3162	0.4743	0.149*	0.50
H8C	0.4349	0.3213	0.4781	0.149*	0.50
H3	0.519 (3)	0.2500	0.042 (2)	0.080*	
H4	0.212 (2)	0.2500	0.259 (3)	0.080*	

### Atomic displacement parameters (Å^2^)

**Table:**

	*U*^11^	*U*^22^	*U*^33^	*U*^12^	*U*^13^	*U*^23^
S1	0.0681 (6)	0.1189 (9)	0.0617 (6)	0.000	−0.0150 (4)	0.000
N1	0.0392 (13)	0.0640 (17)	0.0642 (17)	0.000	0.0028 (12)	0.000
N2	0.0365 (12)	0.0615 (16)	0.0542 (15)	0.000	0.0019 (11)	0.000
N3	0.0416 (13)	0.0775 (18)	0.0508 (16)	0.000	0.0024 (11)	0.000
N4	0.0738 (18)	0.085 (2)	0.0518 (16)	0.000	0.0136 (14)	0.000
C1	0.0369 (14)	0.0618 (19)	0.0503 (17)	0.000	0.0058 (12)	0.000
C2	0.0425 (15)	0.0593 (19)	0.0512 (18)	0.000	0.0011 (13)	0.000
C3	0.0450 (16)	0.074 (2)	0.064 (2)	0.000	−0.0062 (15)	0.000
C4	0.0587 (19)	0.102 (3)	0.0478 (19)	0.000	−0.0054 (16)	0.000
C5	0.0505 (18)	0.097 (3)	0.055 (2)	0.000	0.0091 (15)	0.000
C6	0.0389 (15)	0.078 (2)	0.0579 (19)	0.000	0.0108 (13)	0.000
C7	0.0624 (19)	0.0541 (19)	0.0499 (18)	0.000	0.0033 (15)	0.000
C8	0.126 (3)	0.121 (4)	0.053 (2)	0.000	0.023 (2)	0.000

### Geometric parameters (Å, °)

**Table:**

S1—C7	1.682 (3)	C1—C6	1.460 (4)
N1—C2	1.335 (3)	C2—H2	0.9300
N1—C3	1.340 (3)	C3—C4	1.367 (4)
N2—C6	1.273 (4)	C3—H3A	0.9300
N2—N3	1.382 (3)	C4—C5	1.385 (4)
N3—C7	1.361 (4)	C4—H4A	0.9300
N3—H3	0.899 (10)	C5—H5	0.9300
N4—C7	1.334 (4)	C6—H6	0.9300
N4—C8	1.463 (4)	C8—H8A	0.9600
N4—H4	0.898 (10)	C8—H8B	0.9600
C1—C5	1.379 (4)	C8—H8C	0.9600
C1—C2	1.394 (4)		
			
C2—N1—C3	117.0 (2)	C3—C4—H4A	120.8
C6—N2—N3	117.1 (2)	C5—C4—H4A	120.8
C7—N3—N2	118.9 (2)	C1—C5—C4	119.9 (3)
C7—N3—H3	124 (2)	C1—C5—H5	120.0
N2—N3—H3	117 (2)	C4—C5—H5	120.0
C7—N4—C8	124.7 (3)	N2—C6—C1	121.7 (3)
C7—N4—H4	117 (2)	N2—C6—H6	119.2
C8—N4—H4	119 (2)	C1—C6—H6	119.2
C5—C1—C2	117.0 (2)	N4—C7—N3	114.7 (3)
C5—C1—C6	121.1 (3)	N4—C7—S1	125.2 (2)
C2—C1—C6	121.9 (3)	N3—C7—S1	120.1 (2)
N1—C2—C1	124.1 (2)	N4—C8—H8A	109.5
N1—C2—H2	118.0	N4—C8—H8B	109.5
C1—C2—H2	118.0	H8A—C8—H8B	109.5
N1—C3—C4	123.6 (3)	N4—C8—H8C	109.5
N1—C3—H3A	118.2	H8A—C8—H8C	109.5
C4—C3—H3A	118.2	H8B—C8—H8C	109.5
C3—C4—C5	118.5 (3)		

### Hydrogen-bond geometry (Å, °)

**Table:**

*D*—H···*A*	*D*—H	H···*A*	*D*···*A*	*D*—H···*A*
N4—H4···N2	0.90 (2)	2.14 (3)	2.585 (4)	109 (2)
N3—H3···N1^i^	0.90 (1)	2.09 (1)	2.989 (3)	176 (3)

Symmetry codes: (i) *x*+1, *y*, *z*.
